# Novel Use of Generalizability Theory to Optimize Countermovement Jump Data Collection in Female Athletes

**DOI:** 10.3390/sports13120425

**Published:** 2025-12-02

**Authors:** Alan Huebner, Jonathon R. Lever, Thomas W. Clark, Jonathan D. Hauenstein, John P. Wagle

**Affiliations:** 1 Sports Performance, University of Notre Dame, Notre Dame, IN 46556, USA; alan.huebner.10@nd.edu (A.H.); jwagle@nd.edu (J.P.W.); 2 Department of Applied and Computational Mathematics and Statistics, University of Notre Dame, Notre Dame, IN 46556, USA; tclark23@nd.edu (T.W.C.); hauenstein@nd.edu (J.D.H.)

**Keywords:** CMJ, team sport, testing, monitoring, force plate

## Abstract

Countermovement jump (CMJ) testing is widely used to monitor neuromuscular function, but trial-to-trial reliability depends on the population and testing ecology. Previous reliability prescriptions have often been derived from male cohorts, risking misapplication to female athletes, whose anthropometry, movement strategies, and testing environments differ. This study applied Generalizability Theory (G-Theory) to quantify the within-session reliability of CMJ metrics in NCAA Division I women’s volleyball, softball, soccer, and lacrosse, aiming to isolate the measurement precision independent of day-to-day biological variance. A fully crossed person × trial G-Theory analysis was performed, with the G-study phase estimating variance components and the D-study phase determining the number of trials required to reach actionable dependability (Φ ≥ 0.80). Force–time data from 103 athletes across 282 jumps were analyzed for 14 commonly monitored metrics. Results show that six concentric and takeoff indices, including force at zero velocity, phase-1 concentric impulse, total concentric impulse, jump height, takeoff velocity, and scaled power, achieved Φ ≥ 0.80 from a single trial across all sports. Second-tier variables, such as eccentric duration, phase-2 impulse, and the modified reactive strength index, stabilized within two to three trials, whereas braking impulse, countermovement depth, and deceleration RFD asymmetry required impractical sampling and were deemed fragile (i.e., requiring a greater number of trials to reach acceptable reliability). Compared with the male data, women exhibited larger between-subject variance and higher single-trial dependability for 11 of the 14 studied metrics. Findings support concise, sex-specific trial prescriptions that prioritize stable metrics and minimize unnecessary testing.

## 1. Introduction

Countermovement jump (CMJ) testing on dual force plates is widely used to profile lower-body force production and to monitor neuromuscular status [1,2,3]. Each trial yields phase-specific metrics, spanning eccentric and concentric actions, impulse, velocity, asymmetry, and derived indices [4,5]. Before such metrics can guide monitoring or decisions, their reliability must be established in the context of use. Generalizability Theory (G-Theory), originating with Cronbach and colleagues [6], quantifies reliability by decomposing observed-score variance into design facets (e.g., participant, trial, and session) and their interactions using a random-effects Analysis of Variance (ANOVA) framework. From these variance components, it yields coefficients for relative (Eρ^2^) and absolute (Φ) decisions, allowing investigators to specify how many trials are needed to reach a target reliability under a given design. Since reliability depends on the population and the testing ecology, estimates established in one cohort may not be generalizable to another [7].

Reliability is cohort-dependent rather than a fixed property of an instrument or algorithm [8]. Female athletes differ from male athletes in anthropometric distributions [9,10,11]; neuromuscular strategies during braking and propulsion [12]; injury etiology and the monitoring emphasis placed on asymmetry [13,14,15]; and the practical “ecology” of testing (e.g., session structure, travel, and time constraints for repeated trials) [16]. These factors can shift the relative magnitudes of the person, trial, and residual variance components that underlie G-Theory, altering the number of trials required to achieve a given decision reliability (Φ). Applying reliability prescriptions that have been derived from male cohorts risks misinterpretation, perhaps resulting in over- or understating the required number of trials for female cohorts. A female-specific analysis is, therefore, needed to establish appropriate reliability values and recommended numbers of trials, as well as to assess the extent to which estimates from our previous study transfer to female athletes.

Prior evidence supports this concern but leaves actionable gaps for female athletes. Our previous study quantified the variance components and reliability coefficients for male collegiate athletes across multiple sports, showing that some CMJ metrics stabilize quickly, whereas others demand more trials before Φ reaches commonly used thresholds [17]. Outside of our prior work [17], CMJ reliability studies in female athletes are limited and heterogeneous, often tied to a single device or sport [7,18,19,20], and they are largely analyzed with classical test theory rather than G-Theory. Consequently, trial-count recommendations vary [21], and the stability of asymmetry metrics is inconsistent across contexts [22,23]. As a result, practitioners lack cohort-calibrated trial prescriptions and interpretive criteria for female athletes, especially when multiple facets (person × trial × session) and ecological constraints are considered. In the present study, we therefore replicate the prior design and definitions from our previous study of collegiate male athletes [17], with identical CMJ procedures, the same 14 key metrics, and the same minimum reliability threshold (Φ ≥ 0.80), to enable a direct, cohort-specific comparison of variance structures and trial prescriptions.

Accordingly, this study asks three questions. First, what are the reliability coefficients for key countermovement jump metrics in female athletes under various numbers of trials? Second, how many trials are required to achieve actionable decision reliability (Φ ≥ 0.80) under applied testing constraints? Third, to what extent do reliability coefficient values, and the resulting trial prescriptions, differ from our previous male cohort study to female athletes? By answering these questions, we provide cohort-calibrated guidance on metric selection and trial counts for female athletes, thereby tailoring recommendations and aligning monitoring practice with ecologically valid procedures. This investigation extends our prior male cohort analysis [17] by employing an identical design and measurement framework in female athletes, thereby enabling direct comparison of variance structures and trial requirements across sexes.

## 2. Methods

### 2.1. Subjects

The data was collected on 103 female athletes (age = 20.82 ± 1.78 years; body mass = 70.53 ± 10.60 kg) from four sports within an NCAA Division-I program. The sports in the study included volleyball (VB), softball (SB), women’s soccer (WSOC), and women’s lacrosse (WLAX). These four teams were decided upon for their adequate sample size and adherence to strict data collection protocols, as well as for their diverse range of playing surfaces and movements. The 103 athletes in the sample resulted in 282 total observations in the data, as each athlete recorded two or three trials (the mean number of trials was 2.74; Table 1). While established guidance on appropriate sample sizes for G-theory analyses is scarce, the participant numbers in each sport aligned with or surpassed those reported in prior studies on countermovement jump reliability. Despite the limited sample size guidance for G-theory analyses, the sample sizes per sport are comparable to, or exceed the sample sizes utilized in the existing literature pertaining to the reliability of countermovement jump metrics. Approval of this study was provided by the university’s Institutional Review Board (IRB) (25-07-9455).

### 2.2. Procedures

Data were collected as part of routine day-to-day sports performance support across the academic year and were analyzed retrospectively. Prior to testing, participants completed a standardized warm-up following the RAMP protocol (Raise body temperature and heart rate; Activate key muscle groups; Mobilize major joints; and Potentiate with progressive movements) [24], which was led by a certified strength and conditioning professional. Countermovement jump (CMJ) testing was conducted on dual force platforms (ForceDecks, VALD, Brisbane, Australia) placed on a stable, flat surface and was calibrated in accordance with manufacturer guidelines. Force–time data were captured using the proprietary ForceDecks software (VALD, Brisbane, Australia; version 2.7) at 1000 Hz, and they were processed immediately to derive the discrete variables of interest. For each trial, participants stood on the platforms with feet shoulder-width apart. Following at least one second of quiet standing to ensure accurate measurement of system mass [25], they performed a CMJ on the verbal instruction to jump “as high and as fast as you can” after a countdown of “3, 2, 1, jump.” All jumps were performed with hands on hips, using a self-selected countermovement depth [26], transitioning without pause, and with maximal effort to achieve vertical height in a single continuous action [26]. Each athlete completed a minimum of three individual trials, which were separated by ~5 s of rest and monitored by the practitioner. This interval reflects the established CMJ testing protocols shown to provide reliable measures without inducing fatigue [20]. Force–time curves were then visually inspected to confirm data quality. Per routine collection protocols, trials compromised by technical error (e.g., improper technique and stepping off the platform) were excluded and, if needed, repeated.

We evaluated the fourteen CMJ outcomes commonly adopted in high-performance monitoring that, together, sample discrete phases of the force–time curve and include asymmetry where relevant: countermovement depth (CMd); braking impulse (BI); deceleration rate of force development (DRFD); deceleration RFD asymmetry (DRFDa); eccentric duration (ECCdur); force at zero velocity (F@0v); force at zero velocity asymmetry (F@0va); phase-1 concentric impulse (P1CI); phase-2 concentric impulse (P2CI); total concentric impulse (CI); jump height (JH); takeoff velocity (TOv); scaled power (SP); and modified reactive strength index (RSImod). Operational definitions and units are provided in Table 2. Selection was guided by prevalence in applied practice and coverage of key constructs, eccentric braking and transition characteristics, concentric force/impulse/velocity outputs, and inter-limb asymmetry, supporting athlete monitoring for fatigue management and training adaptation. All variables were computed by the ForceDecks software from 1000 Hz force–time data using the manufacturer’s event-detection procedures and exported as discrete trial-level values for subsequent G- and D-study analyses.

### 2.3. Statistical Analyses

The execution of a reliability analysis via Generalizability Theory can be subdivided into two sequential steps. In the first part, known as the G-study, researchers use ANOVA to estimate the variance components associated with all of the distinct sources of variation in the study design. The data collection in the present study followed a fully crossed person x trial design, since each athlete performed multiple trials. An athlete’s true score (or “universe” score) for any given metric is the value that would be obtained if there were no errors in the measurement process, equivalent in this case to averaging over infinitely many trials on the ForceDecks system for each athlete. Differences in the observed metric values between two athletes on any given trial must, therefore, be attributable to some combination of between-person variance ($\sigma_{p}^{2}$, differences in athletes’ true scores), between-trial variance ($\sigma_{t}^{2}$, systematic differences in scores attributable to trial effects), and residual variance ($\sigma_{pt,e}^{2},$ variation arising from person-by-trial interactions or other unaccounted sources). Using these estimated variance components, the researcher can then calculate the index of dependability, Φ. Also known as the G-coefficient for absolute agreement, Φ returns a value between zero and one, indicating the degree to which the researcher can reliably generalize to the athlete’s true score from a single observed score. In the present study, this would correspond to generalizing to an athlete’s true metric from a single force plate trial. The index of dependability is calculated as the proportion of the total variance caused by differences in the objects of measurement (athletes, in this case). Adhering to the notation from Brennan [27], this is stated mathematically as follows:

$$\Phi =\frac{\sigma_{p}^{2}}{\sigma_{p}^{2}+\sigma_{t}^{2}+\sigma_{pt,e}^{2}}$$

This formula tells us that the reliability of a variable of interest would increase with either an increase in the between-subject variance ($\sigma_{p}^{2}$) or a decrease in one of the other two variance components [6,27]. Practically, an increase in $\sigma_{p}^{2}$ would indicate greater differentiation between athletes in the dataset.

In the current study, however, the researchers did not desire to generalize from a single score but rather, to generalize by averaging over a number of trials instead. This is where the second part of the G-theory framework, known as the D-study, comes into play. The D-study (i.e., “Decision” study) permits the researcher to test out various numbers of trials, raters, occasions, etc., and determine the optimal number for reaching a particular reliability threshold. This is performed by dividing the variance components from the G-study by the desired number of iterations for a given facet, then recalculating the index of dependability, Φ. For example, if the researchers of the current study wished to know the reliability with which they could generalize to a given athlete’s true score by averaging over *n_t_* trials, they would recalculate the between-trial variance as $\sigma_{T}^{2}=\sigma_{t}^{2}/n_{t}$ and the residual variance as $\sigma_{pT,e}^{2}=\sigma_{pt,e}^{2}/n_{t}$.

Again following the notation from Brennan [27], the new index of dependability would then become the following:

$$\Phi =\frac{\sigma_{p}^{2}}{\sigma_{p}^{2}+\sigma_{T}^{2}+\sigma_{pT,e}^{2}}$$

The ability to test out different numbers of iterations for each facet within a given study is what lends Generalizability Theory its great utility with regard to study design, permitting researchers to determine the optimal number of occasions, trials, etc., at which they would collect reliable data without wasting time or resources. Furthermore, when multiple metrics are collected during the same trial, as is the case of the present study’s ForceDecks data collection, D-studies can be simultaneously carried out for all metrics of interest to find the minimum number of trials necessary to obtain reliable data across all metrics.

Therefore, for each of the four women’s sports included in this study, D-studies were performed simultaneously on the 14 kinematic variables of interest in order to determine the optimal number of countermovement jump trials for data collection within each sport. All data processing and statistical analyses were performed using R (version 4.4.0; R Core Team), and all D-studies were executed using the *dtheory* package, a user-built package built on the *gtheory* package.

## 3. Results

Reliability was defined as attaining a coefficient of 0.8 or higher within three or fewer jumps in each sporting cohort [17]. This threshold of 0.8 follows recommendations by Koo and Li (2016) [28], in which a reliability coefficient between 0.75–0.90 is interpreted as “good”. Furthermore, the “three or fewer jumps” clause is a reflection of the researchers’ data collection protocol, with three jumps being the status quo. A metric requiring greater than three jumps would, therefore, necessitate a departure from protocol, without which any conclusions drawn from the data would be untrustworthy.

Table 3 displays the results of the D-study for the volleyball team, which is our first population of interest. The data for this population contained two metrics exhibiting lower reliability, DRFDa and CMd, which would require seven and four jumps, respectively, to achieve the desired reliability threshold of 0.8. Since requiring seven trials from each athlete on every occasion would constitute a waste of training time, DRFDa would almost certainly need to be excluded from the list of routinely collected key metrics in this population due to feasibility. That said, DRFDa may still hold value in targeted contexts (e.g., research or rehabilitation), but its instability renders it impractical as a standard monitoring variable under typical team protocols. Furthermore, regarding CMd, it would be left to the discretion of the analyst to decide whether its statistical importance in building training protocols warrants the execution of one additional trial per athlete (per occasion). If not, then removal of this metric from the population’s list of key metrics would also be required to obtain reliable data across the board, with three jumps becoming the optimal number of trials for future data collections.

The results of the next D-study, this one for the softball team, are shown in Table 4. The G-Theory analysis for this population revealed just one unreliable metric, as DRFDa required eight trials to reach the reliability threshold of 0.8. Upon removal of this variable from the team’s collection of key metrics, the optimal number of jumps for the softball team would be brought down to two. This insight would advocate for a data collection protocol change from the status quo of three trials per occasion, which would free up additional training time for coaches and athletes in this population.

The next D-study was performed on the women’s soccer team, as displayed in Table 5. Similar to the volleyball team, the data from this population of interest contained two unreliable metrics, though this time the inconsistent metrics were BI (nine jumps) and DRFDa (six jumps). Since it would be impractical to ask athletes to jump even six times on the ForceDecks system each time they come in for data collection, these two metrics should be removed from consideration for this particular population. After doing so, the optimal number of jumps for this population would be two, which would again cut out unnecessary data collection and result in additional training and recovery time.

The fourth and final D-study was performed on data from the women’s lacrosse team, and the results are presented in Table 6. Women’s lacrosse was the only population in the study to have zero unreliable metrics. While the optimal number of jumps for this team remained at three, coaches and trainers would have the luxury of consulting all 14 key metrics from the data collection for training strategies and performance optimization.

**Figure 1:**
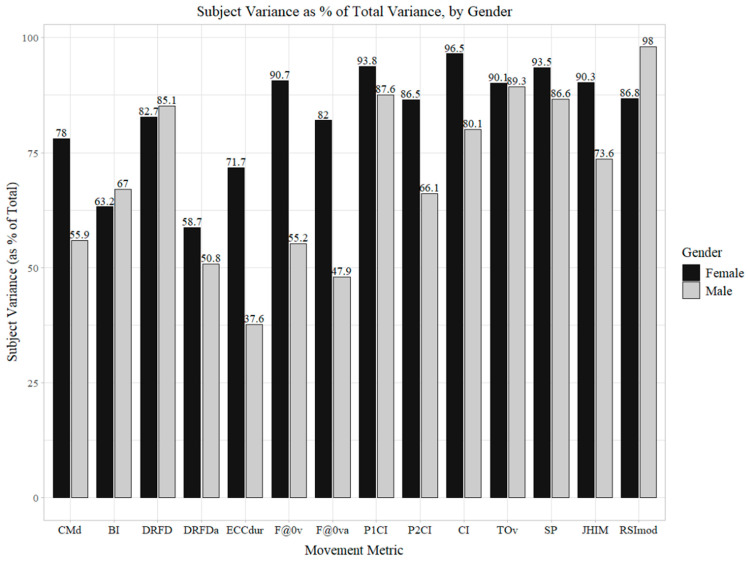
Between-subject variance (**σ**^2^_p_) as a percentage of the total variance in the all-sport G-study, by gender.

Across all four women’s teams, DRFDa was the least reliable metric, requiring about seven, eight, and six trials for VB, SB, and WSOC, respectively, for reliability to reach Φ ≥ 0.80 (Table 7). Cross-cohort variance component patterns are summarized in Figure 1 and interpreted in the Section 4. Specifically, Figure 1 displays the between-subject variance, $\sigma_{p}^{2}$, broken down by female/male (combined over all sports for the current female cohort and a previously studied male cohort) for all 14 key metrics. For example, between-athlete differences in the females accounted for 71.7% of the total variation in ECCdur values, while between-athlete differences in the males accounted for only 37.6% of the total variation in that metric.

## 4. Discussion

The present study evaluated the within-session reliability for 14 CMJ metrics across four NCAA Division I women’s sports using a fully crossed person × trial G-Theory analysis. Principal findings indicate that most of the concentric- and takeoff-derived indices achieved actionable reliability (Φ ≥ 0.80) within three or fewer trials, whereas instability was concentrated in eccentric/braking variables and limb asymmetries (specifically DRFDa, as well as, in sport-specific contexts, BI and CMd. Critically, relative to a previously studied male cohort, the women exhibited larger between-subject variance components ($\sigma_{p}^{2}$) and higher single-trial dependability across most (11 of 14) metrics, indicating that sex-specific protocols were warranted and that male-derived trial prescriptions would tend to overestimate trial requirements and divert attention toward intrinsically fragile metrics (Figure 1). “Fragile” does not mean “uninformative,” rather, it signals that more trials than the status quo of three jumps are generally required for these metrics to reach acceptable levels of reliability. Notably, across all four women’s teams, six key metrics achieved Φ ≥ 0.80 with a single trial, whereas, in our male cohort, only one met this benchmark.

Reliability demonstrated construct-specific patterns consistent with prior CMJ literature, with concentric and takeoff-derived indices stabilizing within a few trials [2,7], whereas braking-phase and asymmetry variables were comparatively unstable [4,5]. In our data, six metrics all met Φ ≥ 0.80 with a single trial: F@0v (0.878–0.920), P1CI (0.903–0.948), CI (0.937–0.971), TOv (0.853–0.925), SP (0.861–0.944), and JH (0.856–0.925). Practically, softball and women’s soccer can operate on two trials after pruning metrics that are infeasible for routine collection are given session constraints (e.g., DRFDa and BI in women’s soccer), while retaining them for targeted use when warranted.

A second tier showed sport-dependent requirements: DRFD often needed two-to-three trials in volleyball and softball but already met Φ ≥ 0.80 in one trial in women’s soccer (Φ_n=1_ = 0.883) and women’s lacrosse (Φ_n=1_ = 0.823); ECCdur generally required two-to-three trials (Φ_n=3_ = 0.860–0.908); F@0va achieved adequate reliability within one-to-three trials depending on sport (Φ_n=1_ = 0.598–0.857; Φ_n=3_ = 0.817–0.947); P2CI reached adequate reliability by three trials in volleyball/softball but was ≥0.80 at one trial in soccer/lacrosse (Φ_n=3_ = 0.895–0.969); and RSImod was ≥0.80 at one trial in volleyball, softball, and soccer but required ~3 trials in lacrosse (Φ_n=3_ = 0.842–0.976). Instability clustered in DRFDa, consistent with the sensitivity of RFD and limb-difference scores to processing and technique variability [12,22], with estimated requirements of ~7 (VB), ~8 (SB), ~6 (WSOC), and ~2 (WLAX) trials to exceed Φ = 0.80; sport-specific instability also appeared in BI for women’s soccer (Φ_n=7_ = 0.775, ≈9 trials) and CMd for volleyball (Φ_n=3_ = 0.780, ≈4 trials), plausibly reflecting self-selected strategy effects [23,26]. A simple stop-rule makes this operational: start Tier-1 metrics at one trial and Tier-2 at two; add one trial only when the most recent values remain inconsistent for that metric; and cap at three to avoid chasing noise.

Asymmetry warrants selective use: F@0va is suitable for routine monitoring within ≤3 trials, whereas DRFDa should be treated as a special-purpose metric (e.g., research or targeted rehabilitation) rather than a standing key metric in team surveillance. By contrast, F@0va achieved adequate reliability within ≤3 trials across teams and was the preferred asymmetry metric under typical session constraints. When RFD asymmetry is clinically indispensable (e.g., targeted return-to-play), stability should be engineered rather than assumed: add averaging only for that metric within a session based on a simple stop-rule tied to that metric, or aggregate it across sessions; otherwise, de-emphasize it in favor of more stable proxies [29,30]. Mechanistically, the reliability hierarchy follows from how each metric samples signal versus noise. Part of this hierarchy is algorithmic, whereby metrics that depend on precise detection of braking onset/offset or on numerical differentiation (e.g., DRFD/DRFDa) are inherently more sensitive to event-detection jitter and filtering than integrative measures (e.g., impulses and TOv). Concentric velocity- and impulse-based indices (e.g., TOv, SP, P1CI/P2CI, and CI) integrate force–time information over broader windows and are less sensitive to small errors in event timing or countermovement depth, yielding higher Φ with fewer trials. Depth and braking measures (CMd, BI) inherit greater strategy variance (self-selected depth and tempo), inflating the residual person × trial/error component. Derivative-heavy constructs, such as DRFD, are additionally penalized because numerical differentiation amplifies high-frequency noise and onset-detection jitter; differencing limbs compounds that uncertainty (DRFDa) [5,22].

At the cohort level, the women sampled exhibited larger between-subject variance components ($\sigma_{p}^{2}$) as a percentage of the total variance for most key metrics (Figure 1), yielding higher Φ at n = 1 trial for 11/14 metrics. These patterns likely reflect a combination of neuromuscular dispersion, sport-specific demands, and testing-ecology factors rather than a singular biological cause. When $\sigma_{p}^{2}$ increases as a proportion of the total variance, Φ also increases because it means that a greater share of the metric variation reflects true score differences rather than differences caused by trial number or person × trial/error noise [6,8]. This may reflect the greater dispersion in the neuromuscular profiles and/or tighter testing ecology in the sampled women. Importantly, relative reliability should be considered alongside absolute interpretation; alerts should be interpreted in the context of the chosen number of trials and the monitoring objectives. Concretely, in women, a further five metrics, DRFD, ECCdur, F@0va, P2CI, and RSImod, typically reached Φ ≥ 0.80 within ≤2 trials on average across sports; by contrast, the male cohort showed four such metrics, highlighting context- and cohort-specific variance patterns rather than strictly sex-driven differences. These differences are visualized in Figure 1 as higher $\sigma_{p}^{2}$ proportions for the women across most key metrics, which directly explains the elevated Φ at n = 1. Compared with the male cohort from our previous study [17], importing male-derived prescriptions would have over-collected trials for many women’s metrics and misdirected effort toward fragile constructs (notably, DRFDa and, sport-specifically, BI). Some overlap existed; for example, TOv was robust in both cohorts, but its divergence in asymmetry and braking-phase stability argues against positing a uniform “three-jumps-for-everything” policy. Sex-specific reliability work is not cosmetic; it changes protocols: prioritize one-trial concentric/takeoff key metric where Φ ≥ 0.80 (F@0v, P1CI, CI, TOv, SP, JH), reserve two-to-three trials for the second tier (DRFD, ECCdur, F@0va, P2CI, and RSImod), and either re-engineer or de-emphasize unstable asymmetry/braking metrics (DRFDa, BI, and CMd). Had male-derived prescriptions been applied wholesale, two errors were likely: (i) unnecessary extra trials on key metrics that would already stabilize quickly in women, and (ii) over-weighting inherently volatile constructs (DRFDa, and BI in women’s soccer), increasing false-positive monitoring flags.

These findings should be interpreted with several caveats. Reliability was estimated within a single-session, fully crossed person × trial design; session-to-session effects were not modeled, so generalization across days or phases was inferred rather than estimated. Trial counts per athlete were modest (mean 2.74), making the D-study projections beyond the observed range (e.g., ≥5–9 trials) tentative. All testing used one hardware–software ecosystem (ForceDecks, VALD) with proprietary event detection; derivative/difference measures (DRFD, DRFDa) were processing-sensitive, and their transferability to other systems may vary. Self-selected depth likely increased strategy variance for CMd and BI; the standardized-depth protocols might have shift variance structures and trial prescriptions. Finally, the cohort came from one NCAA Division I program and several applied covariates (e.g., recent load, travel, menstrual cycle, injury, and fatigue) were not modeled; future work should incorporate a session facet and key covariates, and it should be replicated across devices and competitive contexts. Collectively, these findings support concise, metric-tiered protocols in women’s programs, emphasizing rapidly stabilizing metrics, reserving extra trials for the few metrics that need them, and avoiding routine emphasis on constructs that remained volatile despite added sampling. Sensitivity to a stricter interpretive threshold (e.g., Φ ≈ 0.85) did not alter the metric ranking: the concentric/takeoff indices remained the fastest to stabilize, whereas the braking/asymmetry constructs remained the most fragile, shifting only a subset of second-tier metrics from two to three trials in some sports.

## 5. Practical Applications

These results support a simple, implementable protocol that maps metric stability to trial counts while minimizing testing burden. First, prioritize metrics that achieve Φ ≥ 0.80 with a single trial (i.e., F@0v, P1CI, CI, TOv, SP, and JH) as the core monitoring set on time-constrained days. Second, treat DRFD, ECCdur, F@0va, P2CI, and RSImod as a “second tier”: collect two trials by default, adding a third only when values show atypical spread or inconsistency for that metric. Third, manage fragile or sport-specific liabilities explicitly: avoid DRFDa as a routine metric due to feasibility; if clinically indispensable (e.g., return-to-play), stabilize decisions via metric-specific extra averaging within the session or rolling multi-session means. In women’s soccer, de-emphasize BI as a key metric given its high trial requirement; in volleyball, either standardize CMd or exclude it unless a fourth trial is feasible. Translating this into team protocols: softball and women’s soccer can operationalize two trials after pruning fragile metrics; and volleyball and women’s lacrosse are best supported by three trials (with volleyball reconsidering CMd). Avoid acting on small single-session fluctuations; prioritize replicated changes across the chosen number of trials to reduce false positives and keep athlete management decisions defensible.

## 6. Conclusions

In NCAA Division I women’s sports, most concentric/takeoff CMJ indices were dependable within ≤3 trials, with six key metrics (F@0v, P1CI, CI, TOv, SP, and JH) reaching actionable reliability from a single trial across all four sports. Instability was concentrated in eccentric/braking and asymmetry constructs, especially DRFDa, and—sport-specifically—BI (women’s soccer) and CMd (volleyball). Relative to a previously studied male cohort, women exhibited a larger proportion of between-subject variance ($\sigma_{p}^{2}$) and higher single-trial dependability for 11/14 metrics, meaning that male-derived prescriptions would over-collect trials and misplace emphasis on fragile variables. The practical upshot is clear: sex-specific reliability calibration changes protocols, enabling leaner trial counts, targeted pruning or stabilization of volatile metrics, and decision thresholds tied to specific metrics. Although this study focused on measurement reliability rather than responsiveness, reliability established the essential foundation from which future work can evaluate the sensitivity of CMJ metrics to training load or fatigue-related changes. Accordingly, feasibility considerations argue against including DRFDa, and—sport-specifically—BI (WSOC) and CMd (VB), as routine key metrics; retain them for targeted applications where additional trials or multi-session aggregation are justified. Future work should extend these findings.

## Figures and Tables

**Table 1:** Population included in the analysis.

Sport	Athletes	Observations	Avg. Trials per Athlete	Trial Distribution
Volleyball	19	57	3.00	All athletes had 3 trials
Softball	21	63	3.00	All athletes had 3 trials
Women’s Soccer	21	61	2.91	3 trials n = 19; 2 trials n = 2
Women’s Lacrosse	42	101	2.41	3 trials n = 17; 2 trials n = 25
*Overall*	*103*	*282*	*2.74*	

**Table 2:** Definitions of the countermovement jump metrics included in the analysis.

Metric	Definition
Countermovement Depth (cm)	The maximum negative displacement of the center of mass (CoM) during the eccentric phase of the jump.
Braking Impulse (N·s)	The total impulse generated from the peak negative force to the point at which the CoM velocity reaches zero (end of the eccentric phase).
Deceleration Rate of Force Development (N/s)	The rate of force change calculated from the peak negative velocity to the force value at which the CoM velocity reaches zero (end of the eccentric phase).
Deceleration Rate of Force Development Asymmetry (%)	The percentage difference in deceleration RFD between limbs, calculated as the limb difference divided by the limb sum.
Eccentric Duration (s)	The time elapsed from the onset of movement to the point where the CoM velocity reaches zero (end of the eccentric phase).
Force at Zero Velocity (N)	The force exerted at the point where the CoM velocity reaches zero, calculated using the impulse–momentum relationship.
Force at Zero Velocity Asymmetry (%)	The percentage difference in force at zero velocity between limbs, calculated as the limb difference divided by the limb sum.
P1 Concentric Impulse (N·s)	The impulse generated during the first half of the concentric phase (from zero velocity to the midpoint of the concentric phase).
P2 Concentric Impulse (N·s)	The impulse generated during the second half of the concentric phase (from the midpoint of the concentric phase to triple extension).
Concentric Impulse (N·s)	The total impulse generated from the beginning of the concentric phase (force at zero velocity) to takeoff (when the system mass achieves zero force).
Jump Height (cm)	The maximum vertical displacement of the CoM during flight, estimated using the impulse-momentum method.
Takeoff Velocity (m/s)	The velocity of the CoM at takeoff, estimated using the impulse-momentum method.
Scaled Power (W/kg^2/3^)	The power output near takeoff, calculated as the product of force and time divided by body mass raised to two-thirds power (i.e., allometric scaling).
Reactive Strength Index Modified (AU)	The jump height divided by the total jump duration (sum of the eccentric and concentric durations).

**Table 3:** Dependability coefficient results (Φ) results from the volleyball D-study by trial number.

Metric	n_t_ = 1	n_t_ = 2	n_t_ = 3	n_t_ = 4	n_t_ = 5	n_t_ = 6	n_t_ = 7
Countermovement Depth (CMd)	0.542	0.703	0.780	**0.826**	0.855	0.877	0.892
Braking Impulse (BI)	0.621	0.766	**0.831**	0.868	0.891	0.908	0.920
Eccentric Deceleration RFD (DRFD)	0.714	**0.833**	0.882	0.909	0.926	0.937	0.946
Eccentric Deceleration RFD Asymmetry (DRFDa)	0.398	0.569	0.665	0.726	0.768	0.799	**0.822**
Eccentric Duration (ECCdur)	0.676	**0.807**	0.862	0.893	0.913	0.926	0.936
Force at Zero Velocity (F@0V)	**0.879**	0.935	0.956	0.967	0.973	0.977	0.981
Force at Zero Velocity Asymmetry (F@0Va)	0.598	0.748	**0.817**	0.856	0.881	0.899	0.912
P1 Concentric Impulse (P1CI)	**0.903**	0.949	0.965	0.974	0.979	0.982	0.985
P2 Concentric Impulse (P2CI)	0.740	**0.851**	0.895	0.919	0.934	0.945	0.952
Concentric Impulse (CI)	**0.971**	0.985	0.990	0.993	0.994	0.995	0.996
Takeoff Velocity (TOv)	**0.918**	0.957	0.971	0.978	0.982	0.985	0.987
Scaled Power (SP)	**0.944**	0.971	0.980	0.985	0.988	0.990	0.992
Jump Height Impulse Momentum (JH)	**0.923**	0.960	0.973	0.980	0.984	0.986	0.988
RSI Modified Impulse Momentum (RSImod)	**0.842**	0.914	0.941	0.955	0.964	0.970	0.974

Note. Φ = the dependability coefficient from Generalizability Theory (absolute reliability; range 0–1). Values represent the estimated reliability for each CMJ metric as a function of trial count. Bolded values indicate the instance in which the metric is deemed reliable.

**Table 4:** Dependability coefficient results (Φ) results from the softball D-study by trial number.

Metric	n_t_ = 1	n_t_ = 2	n_t_ = 3	n_t_ = 4	n_t_ = 5	n_t_ = 6	n_t_ = 7
Countermovement Depth	0.709	**0.830**	0.880	0.907	0.924	0.936	0.945
Braking Impulse	0.752	**0.859**	0.901	0.924	0.938	0.948	0.955
Eccentric Deceleration RFD	0.701	**0.824**	0.875	0.904	0.921	0.934	0.943
Eccentric Deceleration RFD Asymmetry	0.358	0.527	0.626	0.691	0.736	0.770	0.796
Eccentric Duration	0.686	**0.814**	0.868	0.897	0.916	0.929	0.939
Force at Zero Velocity	**0.897**	0.946	0.963	0.972	0.978	0.981	0.984
Force at Zero Velocity Asymmetry	**0.800**	0.889	0.923	0.941	0.952	0.960	0.965
P1 Concentric Impulse	**0.937**	0.968	0.978	0.984	0.987	0.989	0.991
P2 Concentric Impulse	0.753	**0.859**	0.901	0.924	0.938	0.948	0.955
Concentric Impulse	**0.967**	0.983	0.989	0.992	0.993	0.994	0.995
Takeoff Velocity	**0.919**	0.958	0.971	0.978	0.983	0.985	0.987
Scaled Power	**0.913**	0.954	0.969	0.977	0.981	0.984	0.987
Jump Height Impulse Momentum	**0.913**	0.955	0.969	0.977	0.981	0.984	0.987
RSI Modified Impulse Momentum	**0.855**	0.922	0.946	0.959	0.967	0.972	0.976

Note. Φ = the dependability coefficient from Generalizability Theory (absolute reliability; range 0–1). Values represent the estimated reliability for each CMJ metric as a function of trial count. Bolded values indicate the instance in which the metric is deemed reliable.

**Table 5:** Dependability coefficient results (Φ) results from the women’s soccer D-study by trial number.

Metric	n_t_ = 1	n_t_ = 2	n_t_ = 3	n_t_ = 4	n_t_ = 5	n_t_ = 6	n_t_ = 7
Countermovement Depth	**0.927**	0.962	0.974	0.981	0.985	0.987	0.989
Braking Impulse	0.329	0.495	0.596	0.663	0.711	0.747	0.775
Eccentric Deceleration RFD	**0.883**	0.938	0.958	0.968	0.974	0.978	0.981
Eccentric Deceleration RFD Asymmetry	0.409	0.581	0.675	0.735	0.776	**0.806**	0.829
Eccentric Duration	0.767	**0.868**	0.908	0.929	0.943	0.952	0.958
Force at Zero Velocity	**0.878**	0.935	0.956	0.966	0.973	0.977	0.981
Force at Zero Velocity Asymmetry	**0.856**	0.923	0.947	0.960	0.968	0.973	0.977
P1 Concentric Impulse	**0.938**	0.968	0.979	0.984	0.987	0.989	0.991
P2 Concentric Impulse	**0.914**	0.955	0.969	0.977	0.981	0.984	0.987
Concentric Impulse	**0.971**	0.985	0.990	0.993	0.994	0.995	0.996
Takeoff Velocity	**0.925**	0.961	0.974	0.980	0.984	0.987	0.989
Scaled Power	**0.940**	0.969	0.979	0.984	0.987	0.989	0.991
Jump Height Impulse Momentum	**0.925**	0.961	0.974	0.980	0.984	0.987	0.989
RSI Modified Impulse Momentum	**0.932**	0.965	0.976	0.982	0.986	0.988	0.990

Note. Φ = the dependability coefficient from Generalizability Theory (absolute reliability; range 0–1). Values represent the estimated reliability for each CMJ metric as a function of trial count. Bolded values indicate the instance in which the metric is deemed reliable.

**Table 6:** Dependability coefficient results (Φ) results from the women’s lacrosse D-study by trial number.

Metric	n_t_ = 1	n_t_ = 2	n_t_ = 3	n_t_ = 4	n_t_ = 5	n_t_ = 6	n_t_ = 7
Countermovement Depth	0.750	**0.857**	0.900	0.923	0.937	0.947	0.954
Braking Impulse	0.659	0.795	**0.853**	0.886	0.906	0.921	0.931
Eccentric Deceleration RFD	**0.823**	0.903	0.933	0.949	0.959	0.965	0.97
Eccentric Deceleration RFD Asymmetry	0.759	**0.863**	0.905	0.927	0.940	0.950	0.957
Eccentric Duration	0.672	**0.804**	0.860	0.891	0.911	0.925	0.935
Force at Zero Velocity	**0.920**	0.958	0.972	0.979	0.983	0.986	0.988
Force at Zero Velocity Asymmetry	**0.857**	0.923	0.947	0.960	0.968	0.973	0.977
P1 Concentric Impulse	**0.948**	0.973	0.982	0.987	0.989	0.991	0.992
P2 Concentric Impulse	**0.859**	0.924	0.948	0.961	0.968	0.973	0.977
Concentric Impulse	**0.937**	0.968	0.978	0.983	0.987	0.989	0.991
Takeoff Velocity	**0.853**	0.920	0.945	0.959	0.967	0.972	0.976
Scaled Power	**0.861**	0.925	0.949	0.961	0.969	0.974	0.977
Jump Height Impulse Momentum	**0.856**	0.922	0.947	0.959	0.967	0.973	0.976
RSI Modified Impulse Momentum	0.639	0.780	**0.842**	0.876	0.899	0.914	0.925

Note. Φ = the dependability coefficient from Generalizability Theory (absolute reliability; range 0–1). Values represent the estimated reliability for each CMJ metric as a function of trial count. Bolded values indicate the instance in which the metric is deemed reliable.

**Table 7:** Summary of the sport-specific trial prescriptions for each key metric.

Metric	Volleyball	Softball	Women’s Soccer	Women’s Lacrosse
Countermovement Depth	4	2	1	2
Braking Impulse	3	2	9	3
Eccentric Deceleration RFD	2	2	1	1
Eccentric Deceleration RFD Asymmetry	7	8	6	2
Eccentric Duration	2	2	2	2
Force at Zero Velocity	1	1	1	1
Force at Zero Velocity Asymmetry	3	1	1	1
P1 Concentric Impulse	1	1	1	1
P2 Concentric Impulse	2	2	1	1
Concentric Impulse	1	1	1	1
Takeoff Velocity	1	1	1	1
Scaled Power	1	1	1	1
Jump Height Impulse Momentum	1	1	1	1
RSI Modified Impulse Momentum	1	1	1	3
	Number of trials required to achieve reliability (Φ ≥ 0.80)			

## Data Availability

Data may be made available upon reasonable request and approval from the institution.
